# Photo Quiz: Victor Babeș

**DOI:** 10.3201/eid3009.231591

**Published:** 2024-09

**Authors:** Andrei Ionut Cucu, Antonio Perciaccante, Raffaella Bianucci

**Affiliations:** Universitatea Stefan cel Mare din Suceava, Iasi, Romania (A.I. Cucu); Spitalul Clinic de Urgență Prof Dr Nicolae Oblu, Iasi (A.I. Cucu);; Azienda Sanitaria Universitaria Giuliano Isontina, Gorizia, Italy (A. Perciaccante);; San Giovanni di Dio Hospital, Gorizia (A. Perciaccante);; University of Turin, Turin, Italy (R. Bianucci);; Université Paris-Saclay, Montigny-le-Bretonneux, France (R. Bianucci)

**Keywords:** *Babesia*, pathology, parasites, vector-borne infections, zoonoses, Victor Babeș

Who is this person?

Here is a clue: He is honored by his name being used for a tickborne protozoan infection.

A) Emile Roux

B) Victor Babeș

C) George Duncan

D) Ronald Ross

E) Walter Reed

See next page for the answer.

This is a photograph of Victor Babeș (1854–1926) (Figure 1), who discovered *Babesia* spp. microorganisms and was a prominent figure in the fields of pathology, virology, and microbiology in Romania (*1*–*3*). Born in Vienna, Austria, on July 28, 1854, he attended high school in Budapest, Hungary, before pursuing his medical studies in Budapest and Vienna. His father, Vincenţiu Babeș (1821–1907), was an Imperial lawyer, teacher, president of the Supreme Court of Hungary, and a founding member of the Romanian Academic Society.

**Figure 1:**
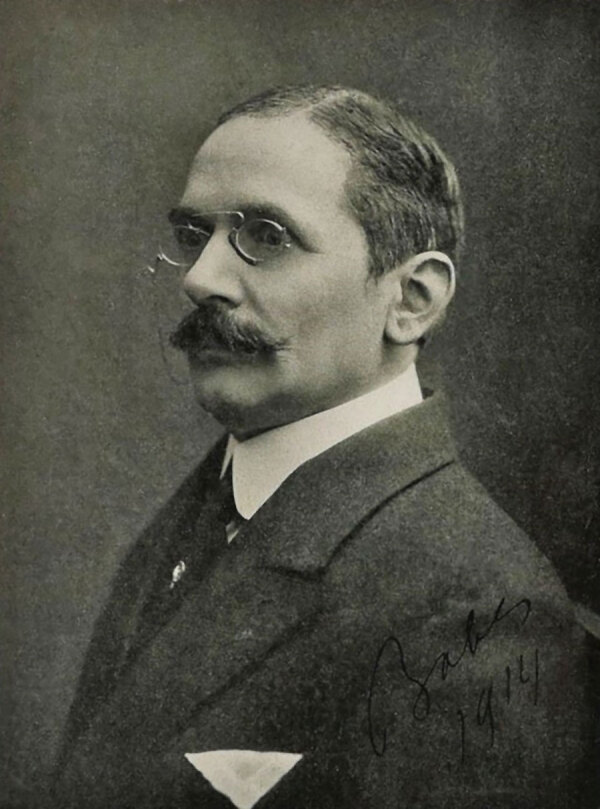
Victor Babeş (1854–1926). Source: University of Kansas Medical Center, Clendening History of Medicine Museum (http://clendening.kumc.edu/dc/pc/babes.jpg).

Victor Babeș began his career when he was 18 years of age in the pathology department at the University of Budapest and later earned his PhD in medical sciences. In 1885, he was appointed as an associate professor at the University of Budapest (*4*). He honed his skills in Europe’s most renowned laboratories, studying with distinguished figures such as Rudolf Virchow (1821–1902), Friedrich Daniel von Recklinghausen (1833–1910), Karl Langer (1819–1887), Justus von Liebig (1803–1873), and Karl von Rockitansky (1804–1878). In the field of microbiology, he was mentored by giants such as Robert Koch (1843–1910) and Louis Pasteur (1822–1895) (*5*–*8*).

One of Dr. Babeș’s greatest achievements was co-authoring the bacteriology treatise *Les bactéries et leur rôle dans l’anatomie et l’histologie pathologiques des maladies infectieuses* with André Victor Cornil (1837–1908); the work is considered a landmark of 19th Century medicine (*9*–*11*). The treatise gained substantial attention; all 3 editions sold out within 5 years. Louis Pasteur suggested that the book should be honored, praise that led the French Academy of Sciences to award the Montyon Prize to the authors (*12*,*13*).

In 1885, Dr. Babeș was appointed as a professor of histopathology in the Faculty of Medicine at the University of Budapest. In 1887, he moved to Bucharest, Romania, where he founded the pathology and bacteriology departments within the Faculty of Medicine at the University of Medicine and Pharmacy Carol Davila Bucharest and was a professor in the bacteriology department until 1926. In 1887, in accordance with the Ministry of Internal Affairs Law no. 1197, the Institute of Bacteriology and Pathology was established in Bucharest; the institute was led by Victor Babeş and now bears his name (Victor Babeş National Institute of Pathology). The Institute is the oldest medical science institute in Romania, initially conceived as a comprehensive medical institute similar to Institut Pasteur in Paris and has pathologic anatomy, bacteriology, veterinary pathology, rabies vaccination, and serology sections.

In 1888, Dr. Babeș established the second center for rabies vaccination in the world, the first having been created by Louis Pasteur in Paris. Furthermore, in 1888, Victor Babeș discovered microorganisms in the erythrocytes of cattle (now known as *B*. *bovis)* and sheep in Romania. He associated the presence of this microorganism with bovine hemoglobinuria, a sign of redwater fever. During 1889–1893, ticks were identified as the transmission vectors for *Babesia* spp. in Texas cattle (*14*). In 1893, the parasites were named *B*. *bovis*, *B*. *ovis*, and *B*. *bigemina*, the genus reflecting the name of their discoverer (*14*). The first case of human babesiosis was diagnosed in 1957 (*14*) in the small town of Strmec, Croatia, not far from Ljubljana, Slovenia.

Dr. Babeș pioneered the understanding of bacterial structures, discovering the metachromatic bodies of the diphtheria bacillus (*Corynebacterium diphtheriae*), which enabled the easiest bacteriologic diagnosis of diphtheria. In 1886, he was the first scientist to report the systemic invasion of organs by the diphteria bacillus and suggested antidiphtheria therapy. He was the first to describe leprosy and tuberculosis bacilli in actinomycotic forms (*2*–*4*,*12*) and demonstrated that some pathogens could be encapsulated and their structures could be observed under the microscope. He also accomplished the staining of microbial cilia.

During 1888–1889, Victor Babeș’ studies laid the experimental and clinical groundwork for serotherapy in medicine, introducing the theory of seroimmunization involving the concurrent introduction of immunizing serum samples prepared with antigenic material in distinct body sections (*4*). In 1889, he published this method in the Annales de l’Institut Pasteur (*11*). In 1895, he formulated the principle of serovaccination. He prepared various serum samples and vaccines in his laboratory for mass prevention of certain communicable diseases. He prepared antidiphtheria serum samples by vaccinating horses and using a method to “offset dormant toxin through blood antitoxins,” which was adopted by several laboratories (*4*,*12*).

Dr. Babeș identified rabies inclusions, which are pathognomonic lesions of rabies found in the cytoplasm of neurons in Ammon's horn of the hippocampus. Rabies inclusions were later rediscovered in 1903 by Aldechi Negri (1876–1912); the inclusions are now known as Babeș-Negri bodies (*8*). Moreover, Dr. Babeș demonstrated experimental rabies virus proliferation in the nerves of humans. In 1887, he observed that heating the rabies virus to 58°C for 2–14 minutes led to controlled attenuation, unlike conventional methods of drying or dilution. Thus, he created a rabies vaccine by heating the virus, which is known as the Romanian technique of immunization.

In 1912, he published the treatise Traité de la rage (published by Librairie J.-B. Baillière et fils in Paris), which received considerable acclaim from the medical scientific community. This extensive treatise on rabies is ≈700 pages long, encompasses all knowledge about rabies at that time, and includes ≈90 works solely on rabies. 

In the field of epidemiology, he initiated a conference on leprosy and pellagra in Romania (*12*) and successfully coordinated fights against 4 cholera epidemics in Europe: Paris (1884), Budapest (1886), Central Europe (1892), and Bulgaria (1893) (*12*,*15*). In 1913, he prepared an anticholera vaccine to combat the cholera epidemic that had broken out among persons in the Army of Romania, who were campaigning in the Second Balkan War in Bulgaria. During 1916–1918, he continued to prepare biological products, remaining in the area occupied by the Central Powers.

In 1893, Victor Babeș became a full member of the Romanian Academy. During his lifetime, he was also appointed an officer of the French Legion d’Honneur and a corresponding member of the Paris Academy of Sciences (*7*).

Victor Babeș died on October 19, 1926, in Bucharest at 72 years of age. In 2012, the postal service of Romania (https://www.posta-romana.ro) issued 2 stamps to commemorate the 125th anniversary of the founding of the Victor Babeş National Institute (Figure 2). His contributions to medicine and science are substantial and should be remembered in the scientific realm of infectious diseases. Through his discoveries, which were published in >1,000 works in many different languages, he opened new horizons in the field of infectious disease pathology.

**Figure 2:**
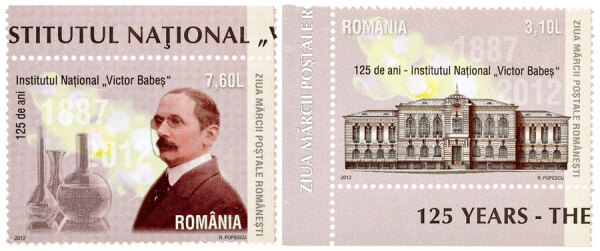
Postage stamps issued in 2012 by the postal service of Romania (https://www.posta-romana.ro) to commemorate the 125th anniversary of the founding of the Victor Babeş National Institute. Source: Romfilatelia. Photography by Will Breedlove, August 2024.

## References

[1] Curcă D. The Romanian scientist: Prof. Victor Babes. Hist Med Vet. 2002;27:333–47. 12146506

[2] Richou R. Victor Babes, his life, his work [in French]. Rev Pathol Gen Physiol Clin. 1964;64:281–2. 14254620

[3] Calciu M. [The 125th anniversary of the birth of V. Babeş (1854-1979). Contribution of Victor Babeş to the advancement of scientific phthisiology] [in Romanian]. Rev Ig Bacteriol Virusol Parazitol Epidemiol Pneumoftiziol Pneumoftiziol. 1979;28:187–91. 229545

[4] Popa MI. Where pathology, microbiology and virology converge: Professor Victor Babeș. Roum Arch Microbiol Immunol. 2021;80:179–88. 10.54044/RAMI.2021.02.08

[5] Babeș MV, Igiroșianu I. Babeș. Oameni de seamă. Bucharest (Romania): Tineretului Publishing House; 1961.

[6] Victor Babeș, pagini alese. Bucharest: Editura de Stat pentru Literatura Științifică; 1954.

[7] Victor Babeș, volum omagial. Bucharest: Editura de Stat pentru Literatura Medicală; 1949.

[8] Babeş V, Babeş M, Horodniceanu F, Nicolau SS. Victor Babeș, opere alese. Bucharest: Editura Academiei Republicii Populare Romîne, 1954.

[9] Cornil AV, Babes V. Les bactéries et leur rôle dans l’anatomie et l’histologie pathologiques des maladies infectieuses: ouvrage contenant les methods speéciales de la bactériologie [in French]. Paris: F. Alcan; 1885.

[10] Stirbu A. [Views of Victor BABES, on the problem of rabies] [in Romanian]. Stud Cercet Inframicrobiol. 1963;14:81–91. 13984189

[11] Rundfeldt C. Drug development: a case study based insight into modern strategies. Rijeka (Croatia): InTech; 2011.

[12] Voinescu D, Mohan A, Constantinescu R, Ciurea AV. Nine decades after the death of the famous scientist Victor Babeş. Proc Rom Acad, Series B. 2016;18:243–50.

[13] Prize Awards of the Paris Academy of Sciences. Prize Awards of the Paris Academy of Sciences. Nature. 1925;115:173–4. 10.1038/115173a0

[14] Uilenberg G. *Babesia*—a historical overview. Vet Parasitol. 2006;138:3–10. 10.1016/j.vetpar.2006.01.035 16513280

[15] Negru I. [Concept of Prof. Victor Babeş of the training and education of the health officer] [in Romanian]. Viata Med Rev Inf Prof Stiint Cadrelor Medii Sanit. 1983;31:211–4. 6417903

